# EXAMINING THE USE AND IMPACT OF CONTRACT CERTIFIED NURSING ASSISTANT STAFFING IN NURSING HOMES

**DOI:** 10.1093/geroni/igad104.1070

**Published:** 2023-12-21

**Authors:** Kezia Scales, Laurie Hailer-O’Keefe, Laura Wagner, Lina Stepick

**Affiliations:** PHI, New York City, New York, United States; UCSF Healthforce Center, San Francisco, California, United States; University of California, San Francisco, San Francisco, California, United States; PHI, New York City, New York, United States

## Abstract

Certified nursing assistants (CNAs) provide the majority of hands-on care in nursing homes, and adequate CNA staffing is associated with better resident and staff outcomes. However, low wages and overall poor job quality make adequate CNA staffing particularly challenging. There is evidence that nursing homes have increased reliance on contract staffing to fill staffing shortages, though the full scope of this trend for CNAs remains unclear and the impacts on care quality are still unknown. This study addresses this gap by examining staffing levels and patterns for fully employed versus contract CNAs in nursing homes over the past five years and assessing how these staffing trends are associated with quality outcomes for nursing home residents. Specifically, this session will present analysis of data from the Centers for Medicare and Medicaid Services (CMS) Payroll-Based Journal daily nurse staffing files, CMS nursing homes data archive, LTCFocus data, community transmission of COVID-19 data, and unemployment data to demonstrate how staffing patterns among fully employed versus contract CNAs varied from 2017 to 2022 according to facility-level characteristics, local market conditions, and pandemic-related factors. We also examine how CNA staffing levels and patterns affected care quality for nursing home residents. These findings will be presented with policy and practice recommendations for strengthening and stabilizing CNA nursing home staffing.

